# Modulation of mRNA Expression of Biomarkers in the UPR-PERK Pathway by Ellagic Acid in Metabolic Dysfunction-Associated Fatty Liver Disease

**DOI:** 10.3390/ijms27104491

**Published:** 2026-05-17

**Authors:** Stephane Pastrana-Cruz, Aarón Domínguez-López, Elizabeth Pérez-Hernández, Ángel Miliar-García, Norma Paniagua-Castro, Laura Adriana Ortiz-León, Antonio Ávila-Guerrero, Raúl J. Delgado-Macuil, Jorge Cornejo-Garrido, María Eugenia Jaramillo-Flores

**Affiliations:** 1 Escuela Nacional de Ciencias Biológicas, Instituto Politécnico Nacional, Mexico City 07738, Mexico; spastranac1900@alumno.ipn.mx (S.P.-C.); npaniagc@ipn.mx (N.P.-C.); adrianaortizleon@gmail.com (L.A.O.-L.); 2 Labotratorio de Biología Molecular, Escuela Superior de Medicina, Instituto Politécnico Nacional, Mexico City 11340, Mexico; adominguezl@ipn.mx (A.D.-L.); amillarg@ipn.mx (Á.M.-G.); aavilag2300@alumno.ipn.mx (A.Á.-G.); 3 Escuela Nacional de Medicina y Homeopatía, Instituto Politécnico Nacional, Mexico City 07320, Mexico; eperezhe@ipn.mx (E.P.-H.); jcornejog@ipn.mx (J.C.-G.); 4 Centro de Investigación en Biotecnología Aplicada, Instituto Politécnico Nacional, Tlaxcala 90700, Mexico; rdelgadom@ipn.mx

**Keywords:** MAFLD, endoplasmic reticulum stress, UPR pathway, ellagic acid

## Abstract

Obesity contributes to an increase in the prevalence of metabolic dysfunction-associated fatty liver disease (MAFLD) and is diagnosed when hepatic steatosis is accompanied by at least one of the following factors: obesity or overweight, diabetes mellitus, or signs of metabolic abnormalities. MAFLD is a term that encompasses a wide range of liver disorders, ranging from simple steatosis to metabolic steatohepatitis, which can progress to cirrhosis and eventually, hepatocellular carcinoma (HCC). Lipotoxicity generated by a high-fat diet causes liver inflammation, therefore, blocking inflammatory pathways is considered a promising strategy to prevent MAFLD progression. Inflammatory responses and oxidative stress are linked to endoplasmic reticulum stress, thereby activating the unfolded protein response (UPR) pathway. Although drugs such as resmetirom and semaglutide have recently been approved for the treatment of MAFLD, there is still a need to identify complementary therapies with different mechanisms of action. In this context, the present study evaluated the hepatoprotective effect of ellagic acid through the modulation of mRNAs of proteins in the UPR-Perk pathway in a murine model fed a high-calorie diet. This study revealed that the high-calorie diet activated the UPR pathway in response to stress, increasing the expression of the *Grp78*, *Eif2ak3*, *Eif2α*, *Ddit3*, *Atf4*, and *Nfe2l2* genes in the liver and epididymal adipose tissue. Ellagic acid modulated the pathway genes and reduced levels of glucose, total cholesterol, HDL and VLDL, triglycerides, insulin, and glycated hemoglobin, and could therefore be considered a hepatoprotective agent.

## 1. Introduction

Multiple risk factors such as overweight, obesity, dyslipidemia, insulin resistance, type 2 diabetes mellitus, and cardiovascular disease contribute to the development of non-alcoholic fatty liver disease (NAFLD), all of which are closely associated with metabolic syndrome. The presence of hepatic fat is clinically relevant, as it can progress to inflammation, fibrosis, cirrhosis, and increase the risk of HCC [1]. NAFLD affects approximately 25% of the adult population worldwide and represents the leading cause of chronic liver disease, as well as one of the most serious complications of obesity [2,3]. The terminology has recently evolved from NAFLD to metabolic dysfunction-associated fatty liver disease (MAFLD), and more recently to metabolic dysfunction-associated steatotic liver disease (MASLD) [4]. MAFLD includes a spectrum ranging from simple steatosis (MAFL) to metabolic dysfunction-associated steatohepatitis (MASH), the latter associated with worse prognosis and increased risk of cirrhosis and cardiovascular mortality [5,6]. It is estimated that 20–25% of patients with MAFLD develop MASH, and approximately 20% of these progress to cirrhosis [7].

Lipotoxicity induced by high-fat diets promotes hepatic inflammation through activation of innate immune pathways and is strongly linked to oxidative stress and endoplasmic reticulum (ER) stress, both of which are key events in MAFLD progression [8]. Excess free fatty acids (FFA), resulting from high-fat diet consumption and insulin resistance, lead to increased hepatic lipid uptake and intrahepatic triglyceride accumulation. This overload impairs mitochondrial β-oxidation and promotes alternative oxidative pathways, increasing the production of ROS and creating a proinflammatory environment [9]. Additionally, adipose tissue dysfunction contributes to ER stress through increased lipid storage, lipogenesis, and adipokine secretion [10]. Elevated circulating FFA and dysfunctional adipose tissue further exacerbate hepatic steatosis, inflammation, and cellular damage through dysregulation of genes involved in metabolic homeostasis [11].

ER stress activates the UPR, a cellular mechanism aimed at restoring protein-folding homeostasis. The UPR is mediated by three main transmembrane sensors: inositol-requiring enzyme 1 (IRE1), activating transcription factor 6 (ATF6), and protein kinase R-like ER kinase (PERK). Upon activation, PERK phosphorylates eukaryotic initiation factor 2α (eIF2α), regulating protein synthesis at the post-transcriptional level [12,13]. The key role of eIF2α in hepatic steatosis lies in its ability to translate an endoplasmic reticulum (ER) stress signal into a genetic survival response [14]. The kinase PERK, in the presence of proteotoxic stress caused by lipid accumulation, activates and catalyzes the incorporation of a phosphate group into the Ser51 residue of eIF2α to phosphorylate it. This post-translational modification alters eIF2α’s affinity for initiation factors, leading to a translation paradox: it allows for the specific translation of the transcription factor ATF4 while simultaneously suppressing global protein synthesis to reduce the ER’s workload. After being synthesized, ATF4 moves to the nucleus. There, it acts as the primary effector of the eIF2α/ATF4 axis, which is directly responsible for regulating the transcription of key genes of the autophagy machinery. In particular, it controls the levels of the messenger RNA (mRNA) for MAP1LC3B (LC3-II) and ATG5. The cell cannot generate the elements required to initiate autophagy if this molecular mutation at Ser51 does not occur, and as a result, ATF4 is not activated.

Although the UPR initially promotes cell survival, its chronic activation contributes to inflammation, steatosis, and fibrosis, playing a central role in liver disease progression [15].

Oxidative stress, defined as an imbalance between ROS production and antioxidant defenses, is a key factor in MAFLD pathogenesis [16,17]. This imbalance triggers adaptive cellular responses, including the activation of antioxidant enzymes and transcription factors to restore redox homeostasis [18]. However, sustained oxidative stress exacerbates liver injury and promotes disease progression [19,20]. Therefore, antioxidant-based interventions have gained attention as potential therapeutic strategies.

Natural compounds rich in polyphenols have been widely studied due to their antioxidant, anti-inflammatory, and metabolic regulatory properties. Among them, ellagic acid has demonstrated hepatoprotective effects, including modulation of oxidative stress, inflammation, and lipid metabolism [21,22]. Previous studies have primarily focused on its ability to activate the Nrf2 pathway and inhibit lipogenic and inflammatory signaling pathways, such as NF-κB and MAPK, thereby reducing hepatic steatosis [19]. However, its effects on the PERK branch of the UPR remain poorly understood.

Therefore, this study aims to evaluate the hepatoprotective effects of ellagic acid through modulation of the UPR-PERK pathway, analyzing both gene and protein expression of key mediators (GRP78, PERK, eIF2α, ATF4, CHOP, and NRF2), as well as metabolic parameters associated with obesity and MAFLD induced by a high-calorie diet. To our knowledge, this is the first study to simultaneously assess transcriptional and protein-level changes in PERK-related signaling under ellagic acid intervention, providing novel insights into its role in ER stress modulation in MAFLD.

## 2. Results

During the experimental period, weekly water intake was monitored in the standard control group (SD), the high-calorie diet (HCD) group, and the HCD group treated with ellagic acid (HCD + EA).

**Figure 1:**
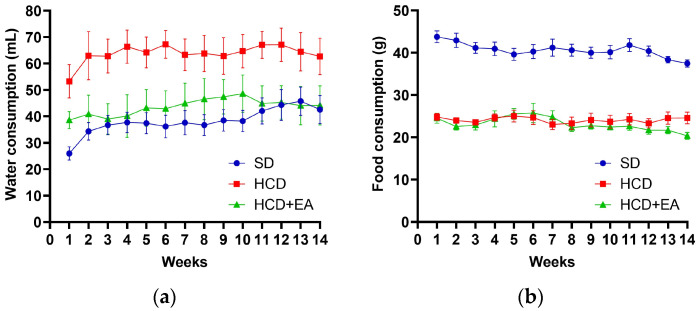

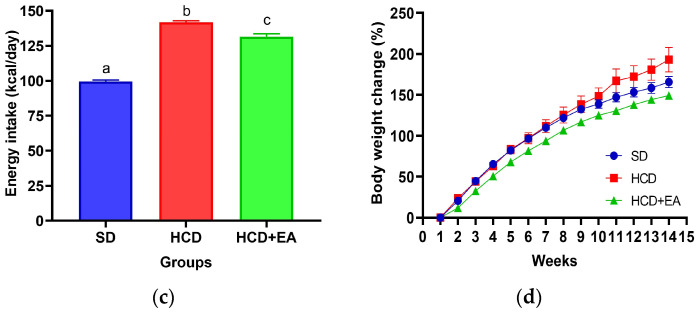
Metabolic parameters during experimental treatment: water intake, food intake, energy intake, and body weight change. (**a**) Water intake (mL), (**b**) food intake and (**d**) body weight change (%) were monitored weekly during the experimental period. These variables were analyzed using two-way ANOVA with repeated measures (group × time), followed by Tukey’s post hoc test. (**c**) Energy intake (kcal/day) was analyzed using one-way ANOVA followed by Tukey’s post hoc test. The groups correspond to standard diet (SD), high-calorie diet (HCD) and Ellagic acid treatment (HCD + EA) under high-calorie diet. Data are expressed as mean ± SEM, *n* = 8. Different letters indicate statistically significant differences between groups (*p* ≤ 0.05).

The HCD group exhibited higher water consumption compared to the SD group, while the HCD + EA group showed a significant difference only in weeks 1, 3, and 6. The HCD + EA group showed water and fructose consumption similar to that consumed by the SD group (Figure 1a).

Food consumption throughout the 14-week experiment showed that the group fed the standard diet ingested more food than the HCD and HCD + EA groups. The consumption of the HCD and HCD + EA groups fed the high-calorie diet was very similar during the 14 weeks (Figure 1b).

Daily energy consumption per animal was calculated from the water and food intake data. It can be seen that the HCD group maintained a higher energy consumption than the SD group, while the energy consumption of the HCD + EA group showed a tendency to decrease compared to the HCD group. However, considering that the SD group had a higher food intake throughout the trial period due to its low-calorie diet, this group had a lower energy consumption (Figure 1c).

The weight gain of the three groups showed an upward trend, with the HCD group outperforming the SD group. The HDC + EA group showed a downward trend compared to the HCD group and had a similar weight to the SD group (Figure 1d).

### 2.1. Lipid and Biochemical Profile

Table 1 shows that the group fed a high-calorie diet (HCD) showed greater weight gain compared to the SD group, and this difference continued to be progressively higher until the end of the study. Meanwhile, the group treated with ellagic acid (HCD + EA) reduced weight gain compared to the HCD group at the end of the 14-week treatment. The HCD group showed a weight gain of 18% over the SD group, and the HCD + EA group only increased by 7% over the SD group.

Regarding energy consumption, the HCD group had a higher caloric intake than the SD group, while the HCD + EA group had a lower caloric intake than the HCD group.

The HCD group showed an increase in the biochemical profile in glucose, insulin, and glycated hemoglobin levels. There was also an increase in the lipid profile: total cholesterol, LDL cholesterol, VLDL cholesterol, and triglycerides, with the latter increasing by approximately 90% over the SD group, while HDL cholesterol decreased. The HCD + EA group showed a reduction compared to the HCD group in the biochemical profile in glucose, insulin, and glycated hemoglobin levels, while in the lipid profile, total cholesterol, VLDL cholesterol, and triglyceride levels were reduced, but in the case of HDL cholesterol, it remained at similar levels to the other groups, and LDL cholesterol increased.

**Figure 2:**
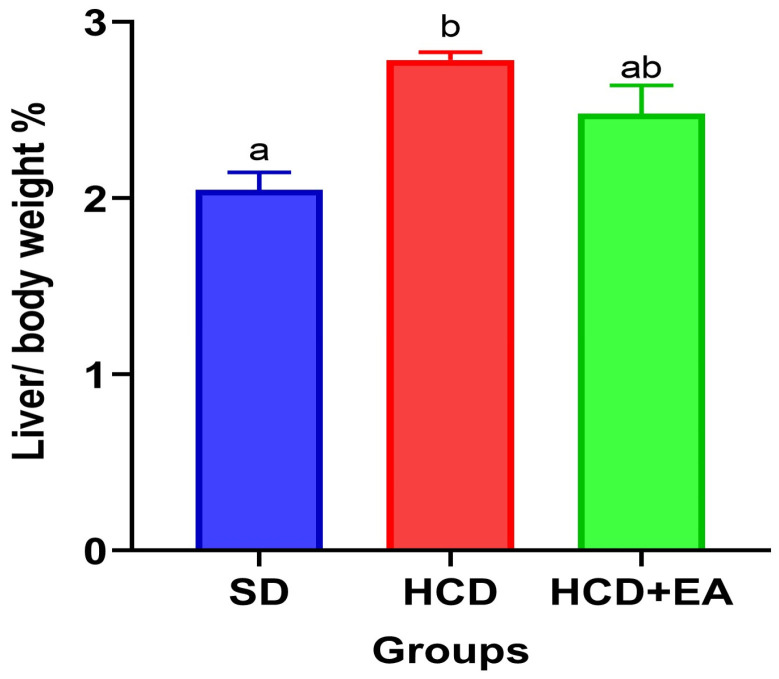
Effect of ellagic acid treatment on the hepatic index. The liver-to-body weight ratio (Liver/BW%) was calculated for each group to evaluate changes in liver size associated with the hypercaloric diet. The groups correspond to standard diet (SD), high-calorie diet (HCD), and high-calorie diet with ellagic acid treatment (HCD + EA). Data are expressed as mean ± SEM (*n* = 8) and were analyzed using one-way ANOVA with Tukey’s post hoc test (*p* ≤ 0.05). Different letters indicate statistically significant differences between groups.

Figure 2 shows the liver-to-body-weight ratio (liver/BW %) expressed as a percentage of liver weight relative to total body weight. The HCD group showed a significant increase in liver/BW % compared to the S group.

**Figure 3:**
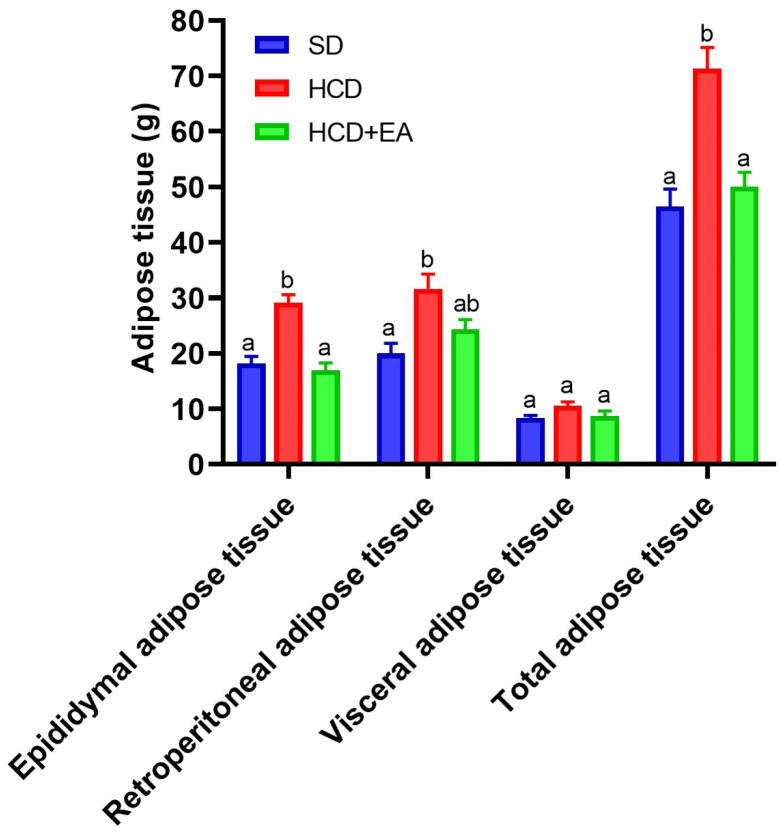
Effect of ellagic acid on adipose tissue accumulation in a high-calorie diet model. Adipose tissue weight (epididymal, retroperitoneal, visceral, and total) was measured in the SD, HCD, and HCD + EA groups. Data are expressed as mean ± SEM (*n* = 8). Statistical analysis was performed using one-way ANOVA followed by Tukey’s post hoc test. Different letters indicate significant differences between groups (*p* < 0.05).

Figure 3 shows the total adipose tissue weight gained by the different experimental groups. A greater increase is seen in the HCD group, while the HCD + EA group showed a decrease in adipose tissue production. The most relevant changes are seen in the epididymal and retroperitoneal adipose tissue, where ellagic acid produces a reduction in the increase, especially in the epididymal adipose tissue.

### 2.2. Histological Analysis of Liver Tissue

**Figure 4:**
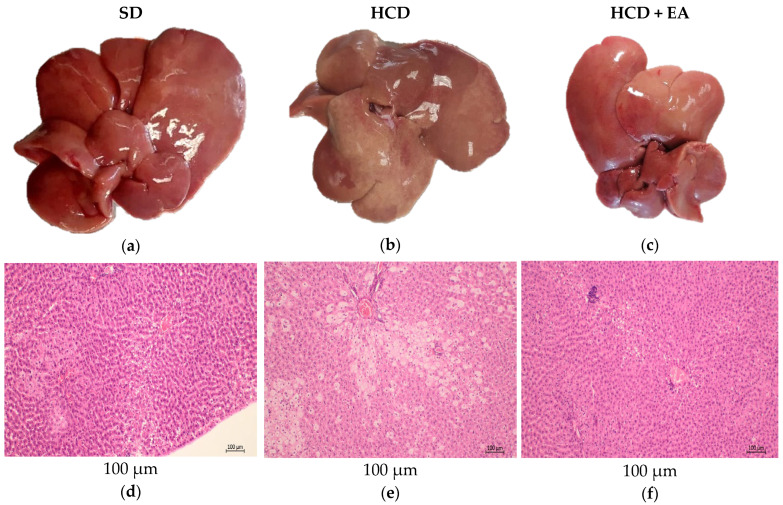

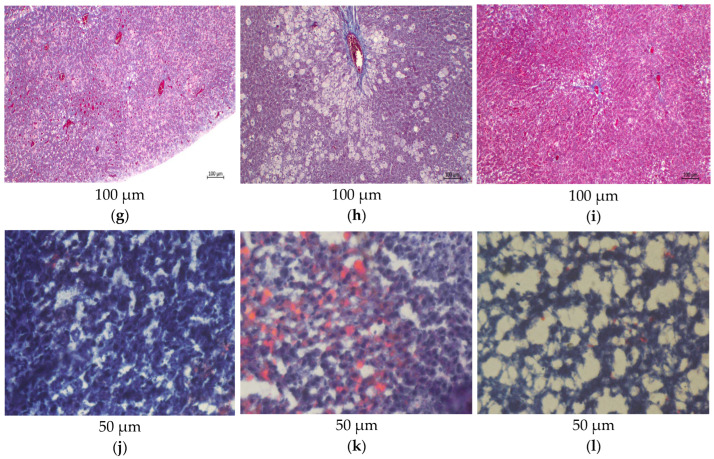
Macroscopic and histological evaluation of liver tissue in a high-calorie diet model treated with ellagic acid. (**a**–**c**). Representative macroscopic images of livers from the standard diet (SD) group, the high-calorie diet (HCD) group, and the ellagic acid (HCD + EA) treatment group, respectively. Images (**a**–**c**) of liver tissue are shown for comparison of hepatic morphology; variations in color and surface appearance are also evident in these images. Figures (**d**–**f**). Representative liver sections stained with hematoxylin and eosin (H&E) 100× showing the general hepatic architecture. The SD group exhibits preserved tissue organization, with hepatocytes arranged in regular cords and a normal sinusoidal structure (arrowheads indicate hepatocytes; arrows indicate sinusoids). In contrast, the HCD group shows hepatocellular alterations, including cytoplasmic vacuolization and disruption of hepatic architecture. The dotted circle indicates the centrilobular vein, and the stars indicate cytoplasmic vacuoles. The HCD + EA group shows partial preservation of the hepatic structure, with reduced vacuolization compared to the HCD group. (**g**–**i**). Masson’s trichrome (100×) stain illustrating the distribution of the extracellular matrix. No clear evidence of fibrosis was observed in any group, although mild alterations in tissue organization were detected in the HCD group. Figures (**j**–**l**). Figures (**j**–**l**) show the results of the Oil Red V (40×) staining technique, which reveal lipid accumulation in each of the experimental groups.

Figure 4 shows the macroscopic and microscopic appearance of the liver at week 14. Macroscopically, no clear differences in liver size were observed between the groups, although slight variations in color and surface appearance were noted (Figure 4a–c). The SD group presented a homogeneous reddish-brown coloration with no evident alterations in macroscopic morphology and a weight of 12.53 ± 0.5 g (Figure 4a), the HCD group showed a liver with a whitish coloration and a weight of 17.92 ± 0.4 g (Figure 4b), and the HCD + EA group showed a coloration similar to that of the SD group, with a weight of 14.97 ± 0.5 g (Figure 4c). Histological analysis using hematoxylin and eosin (H&E) staining revealed that the hepatic architecture was preserved in the SD group; in this image, micro- and macrovesicular lipid changes are observed on the right side, and predominantly microvesicular changes on the left side, with hepatocytes arranged in regular cords and no signs of necrosis or inflammatory infiltrate (Figure 4d). The HCD group showed marked microvesicular fatty changes, with periportal, mediolobular, and centrolobular distribution, which is associated with the development of steatosis (Figure 4e). The HCD + EA group showed two small clusters of lymphocytes, one with a centrolobular distribution and the other with a mediolobular distribution, in addition to mild microvesicular and macrovesicular fatty changes (Figure 4f).

Masson’s trichrome staining confirmed the absence of fibrosis (Figure 4g) in the SD group. In contrast, the HCD group showed no clear evidence of advanced fibrosis, although mild changes in the distribution of the extracellular matrix were observed (Figure 4h). The group treated with ellagic acid showed no evident fibrosis, with a pattern similar to that observed in the control group (Figure 4i).

Oil red V staining revealed minimal lipid accumulation in the SD group (Figure 4j). In contrast, the HCD group showed a marked increase in lipid deposition, evidenced by intense red staining and the presence of lipid droplets within hepatocytes (Figure 4k). The group treated with HCD + EA showed a reduction in lipid accumulation compared to the HCD group, indicating a partial attenuation of hepatic steatosis (Figure 4l).

### 2.3. Effect of Ellagic Acid on Biomarkers of the UPR Signaling Pathway in Liver Tissue

**Figure 5:**
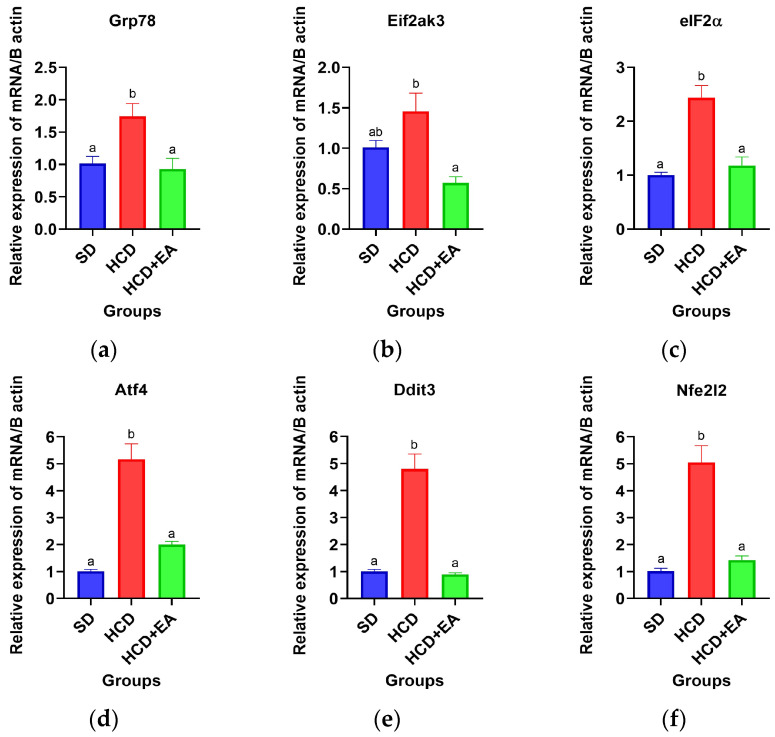
Effect of ellagic acid on the mRNA expression of UPR pathway biomarkers in liver tissue. (**a**) *Grp78* gene expression; (**b**) *Eif2ak3* gene expression; (**c**) *eIF2α* gene expression; (**d**) *Atf4* gene expression; (**e**) *Ddit3* gene expression; (**f**) *Nfe2l2* gene expression. Values are presented as mean ± SEM. mRNA expression was compared using one-way ANOVA (*p* ≤ 0.05). Different letters indicate statistically significant differences between groups.

Figure 5 shows the modulation due to HCD and ellagic acid treatment of the expression of mRNA biomarkers of the UPR-Perk pathway in liver tissue. The HCD group showed increased expression of *Grp78*, *Eif2ak3*, *Eif2α*, *Atf4*, *Ddit3*, and *Nfe2l2* compared to the SD group, indicating activation of endoplasmic reticulum stress signaling under high-calorie diet conditions. *Grp78* expression was significantly increased in the HCD group compared to the SD group, while ellagic acid treatment restored expression levels to values similar to those of the SD group (Figure 5a). Similarly, *Eif2ak3* expression increased in the HCD group, whereas the HCD + EA group showed significantly lower expression than the HCD group (Figure 5b). A marked increase in *Eif2α* expression was observed in the HCD group compared to the SD group, while the HCD + EA group showed a significant reduction in its expression (Figure 5c). Similarly, *Atf4* and *Ddit3* expression levels increased markedly in the HCD group, whereas the HCD + EA group showed significant decreases in both genes, approaching the levels of the SD group (Figure 5d,e). *Nfe2l2* expression was also significantly higher in the HCD group than in the SD group. However, the HCD + EA group reduced *Nfe2l2* expression compared to the HCD group (Figure 5f). This decrease could reflect reduced oxidative stress and a reduced need for compensatory antioxidant signaling following the restoration of redox homeostasis. These findings suggest that the high-calorie diet induced activation of the UPR-Perk pathway, while treatment with ellagic acid attenuated the expression of mediators related to oxidative stress and endoplasmic reticulum.

**Figure 6:**
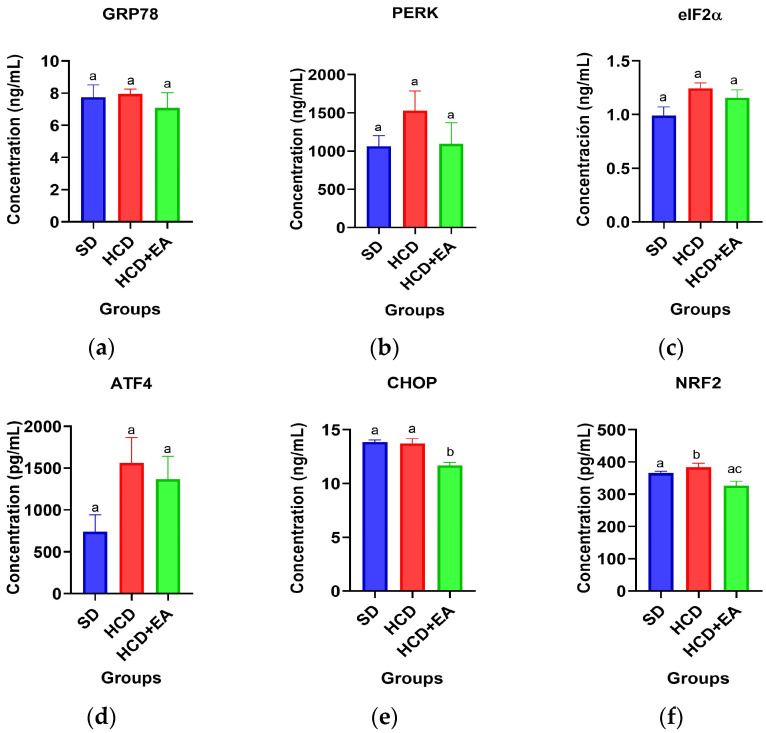
ELISA quantification of proteins involved in the UPR-Perk pathway in liver tissue. Standard diet (SD), high-calorie diet (HCD), and high-calorie diet with ellagic acid (HCD + EA) treatment. (**a**) Quantification of GRP78 protein; (**b**) Quantification of PERK protein; (**c**) Quantification of eIF2α protein; (**d**) Quantification of ATF4 protein; (**e**) Quantification of CHOP protein; (**f**) Quantification of NRF2 protein. Values are presented as mean ± SEM, and statistical analysis was performed using one-way ANOVA (*p* ≤ 0.05). Different letters indicate statistically significant differences between groups.

GRP78 protein expression, which reveals that the HCD group shows an upward trend compared to the SD group, while the HCD + EA group shows a decrease in expression compared to the HCD group and is even lower than the SD group (*p* > 0.05) (Figure 6a). Expression of the PERK protein shows a tendency to increase in the HCD group compared to the SD group. In contrast, the HCD + EA group shows a tendency to decrease compared to the HCD group and is even similar to the SD group (*p* < 0.05) (Figure 6b). Figure 6c shows the data for eIF2α protein expression, indicating that the HCD groups exhibited an increasing trend compared to the SD group, whereas the HCD + EA group showed a decrease in expression compared to the HCD group (*p* > 0.05). The ATF4 protein expression, which shows that the HCD group tends to increase compared to the SD group, while the HCD + EA group shows a decrease compared to the HCD group (*p* > 0.05) (Figure 6d). CHOP protein expression, showing a significant difference between the HCD + EA group and the HCD and SD groups, as it shows a tendency to decrease its expression levels and therefore a lower protein concentration (*p* < 0.001) (Figure 6e). Figure 6f shows the results for the NRF2 protein, indicating that the HCD group tends to increase compared to the SD group, while the HCD + EA group decreased compared to the DHC group, and even decreased more than the SD group (*p* < 0.01).

Ellagic acid treatment effects on the activity of enzymes involved in maintaining redox homeostasis, levels of non-enzymatic antioxidants, and products of protein and lipid peroxidation were measured. Antioxidant capacity was assessed using the ORAC assay, which revealed significant differences among the three experimental groups. The HCD group showed a downward trend (241.9 ± 9.39, *p* < 0.05), which is related to the high-fat diet, as this generates oxidative stress, producing reactive ROS that negatively affect redox homeostasis. On the other hand, the HCD + EA group showed a significant increase in antioxidant capacity (295.7 ± 12.84), reaching levels comparable to those of the SD group. This suggests that treatment with ellagic acid acts as an antioxidant agent or intervenes in cellular mechanisms that promote the restoration of redox balance (Table 2). Analysis of enzyme activity showed that superoxide dismutase (SOD) activity was lower in the HCD group (0.117 ± 0.07) compared to the SD group (0.132 ± 0.07), although no statistically significant difference was observed. On the other hand, the HCD + EA group showed a tendency toward an increase (0.220 ± 0.02) compared to the HCD group. This increase in SOD activity in the HCD + EA group indicates significant antioxidant activation, as SOD is the first line of defense against the superoxide radical, suggesting that the treatment promotes the initial elimination of reactive oxygen species. Catalase decreased in the HCD group (0.008 ± 0.002) compared to the SD group (0.012 ± 0.01) and increased in the HCD + EA group (0.025 ± 0.01). This indicates that the HCD group has a lower capacity to degrade hydrogen peroxide. The simultaneous increase in SOD and CAT is consistent, as SOD generates H_2_O_2_, which CAT eliminates.

GPx activity was significantly lower in the HCD group compared to the SD group, while in the HCD + EA group it showed a tendency to increase compared to the HCD group. Regarding the parameters of the non-enzymatic antioxidant system, GSSG levels showed a tendency to increase in the HCD group compared to the SD group (*p* = 0.08), while the HCD + EA group showed a tendency to decrease compared to the HCD group. Contrary to these results, for GSH levels, the HCD group showed a tendency to decrease compared to the SD group, while the HCD + EA group showed a tendency to increase compared to the HCD group (*p* > 0.05). These results led to an increase in the redox index in the HCD group.

Regarding lipid peroxidation products, a significant increase in MDA content was observed in the HCD group compared to the SD group, while the HCD + EA group showed an important decrease (42.3%) compared to the HCD group. Aldehyde dehydrogenase (ALDH) activity showed no significant changes between the HCD group (0.75 ± 0.34, *p* < 0.05) and the SD group (0.44 ± 0.20). In contrast, the ellagic acid (HCD + EA)-treated group showed a significant increase in ALDH activity (2.617 ± 0.58) compared to the other two groups. ALDH plays a crucial role in the detoxification of toxic aldehydes derived from lipid metabolism and oxidative stress, suggesting that its activation may represent a protective mechanism.

## 3. Discussion

Obesity is closely linked to liver disorders and is considered a key etiological factor in the development of fatty liver disease. Metabolic abnormalities associated with obesity promote inflammatory responses and oxidative stress. In the present study, the HCD group showed an increase in body weight compared to the standard group (SD), confirming the metabolic impact of this dietary model. Interestingly, the administration of ellagic acid (HCD + EA) attenuated this increase, suggesting a modulatory effect on body weight gain. A moderate reduction in body weight has been associated with a lower risk of chronic diseases, such as cardiovascular disease, hypertension, and type 2 diabetes [23].

In relation to weight gain, the rats receiving the high-calorie diet reduce the amount of food consumed compared to the SD group. This difference can be attributed to the energy density of each diet: the standard diet contains fewer calories per gram, forcing the animals to consume a greater volume to meet their energy needs, while the high-fat diet, with its higher calorie content, allows the same requirement to be met with a lower food intake.

The HCD group showed an increase in glucose, insulin, and glycated hemoglobin levels compared to the SD group, making it likely that this group is insulin resistant, while the HCD + EA group showed a reduction in these parameters, indicating an improvement in metabolic homeostasis. Adipose tissue plays a central role in lipid storage; however, excessive accumulation leads to lipotoxicity, which promotes inflammation, oxidative stress, and insulin resistance [24].

Regarding the lipid profile, the HCD group showed an increasing trend in total cholesterol, LDL cholesterol and triglycerides compared to the SD group. These alterations are associated with an increased risk of cardiovascular disease, as LDL and VLDL lipoproteins contribute to atherogenesis, whereas HDL exerts protective effects [25]. Although these changes did not reach statistical significance in all parameters, they reflect a pattern consistent with metabolic dysregulation.

It is essential to explain the approach of the current study. The experimental design used is a model for the prevention and mitigation of MAFLD caused by a high-calorie diet, in which ellagic acid was administered concurrently with the diet to examine its potential to modify the molecular processes leading to liver damage associated with chronic lipotoxicity. This perspective is part of an approach that studies bioactive compounds and their potential to interfere with the pathophysiological cascade from early stages, before liver damage becomes irreversible. This design differs from a treatment model in which the MAFLD condition is first established and then the intervention begins.

Histological analysis revealed clear differences between experimental groups. The SD group showed preserved hepatic architecture, whereas the HCD group exhibited macrovesicular vacuolization consistent with hepatic steatosis, reflecting lipid accumulation in hepatocytes. These changes are associated with increased free fatty acid flux, enhanced de novo lipogenesis and reduced mitochondrial β-oxidation, leading to oxidative stress [26]. Notably, no clear evidence of fibrosis was observed, suggesting that the model corresponds to an early or moderate stage of liver damage.

Interestingly, despite the histological evidence of steatosis, no significant increases in AST or ALT levels were observed in the HCD group. This finding is consistent with previous reports indicating that transaminase levels may remain within normal ranges during early or moderate stages of NAFLD, even in the presence of hepatic lipid accumulation [5]. Moreover, marked elevations in A ST and ALT are more commonly associated with advanced hepatocellular injury and apoptosis rather than simple steatosis [27]. Therefore, the absence of significant transaminase alterations in the present study may reflect the moderate stage of liver injury induced by the HCD model.

The HCD + EA group showed reduced hepatocellular vacuolization and improved tissue organization compared to the HCD group, suggesting a protective effect against diet- induced hepatic alterations.

Although AST and ALT values in the HCD + EA group were higher than those observed in the HCD group, these values remained within physiological ranges reported for Wistar rats and were not accompanied by histological evidence of severe hepatocellular damage or fibrosis. In addition, ellagic acid treatment improved oxidative stress parameters and attenuated the expression of PERK-associated ER stress mediators, supporting a protective rather than hepatotoxic effect [27,28].

This improvement may be associated with the antioxidant properties of ellagic acid. In the present study, HCD + EA increased GPx activity, while GSH levels did not show significant changes. Additionally, a reduction in malondialdehyde (MDA) levels was observed, indicating decreased lipid peroxidation and improved control of the intracellular redox state, consistent with previous reports [29]. The lipid accumulation in liver tissue consists primarily in triglycerides [30]. The accumulation of excess lipids triggers oxidative stress, damages mitochondria, and leads to chronic inflammation, thereby facilitating the progression of NAFLD. Mitochondrial overload impairs β-oxidation capacity and increases ROS, exacerbating liver injury. This oxidative stress leads to the formation of malondialdehyde (MDA), a byproduct of lipid peroxidation and a marker of cellular damage [31].

ROS play a central role in the progression of hepatic steatosis and metabolic liver damage. Under hypercaloric conditions, excessive lipid accumulation promotes mitochondrial dysfunction, endoplasmic reticulum stress, and activation of ROS-producing systems, leading to oxidative damage and lipid peroxidation. In the present study, the HCD group showed increased MDA levels and impaired antioxidant balance, indicating elevated oxidative stress. In contrast, EA treatment increased the activity of antioxidant enzymes such as SOD, CAT, and GPx while reducing lipid peroxidation. These effects may be associated with the polyphenolic structure of ellagic acid, which enables direct ROS-scavenging activity and contributes to restoration of redox homeostasis.

Glutathione peroxidase (GPx) is one of the body’s most potent antioxidant defenses. It protects cells from damage caused by free radicals, specifically lipid peroxides and hydrogen peroxide, by reducing them to alcohol and water, respectively, which can then be further detoxified and removed from the body or, in the case of water, utilized as needed [30].

In this study, the HCD group showed increased oxidative stress, as evidenced by elevated MDA levels and alterations in antioxidant defenses. In contrast, HCD + EA treatment enhanced antioxidant enzyme activity, including SOD, CAT and GPx, while reducing MDA levels. These findings suggest that ellagic acid improves redox balance primarily through modulation of the enzymatic antioxidant system rather than changes in glutathione levels.

Ellagic acid is a polyphenolic compound with multiple hydroxyl groups that enable it to scavenge reactive oxygen species and reactive nitrogen species, contributing to its antioxidant capacity. The observed improvement may be related to the antioxidant properties of ellagic acid, which modulates the glutathione system and antioxidant defenses. In the present study, ellagic acid did not significantly alter GSH levels, while changes in GPx activity and the reduction in MDA content suggest a decrease in lipid peroxidation and improved control of the intracellular redox state. These findings are consistent with previous reports indicating that ellagic acid can enhance antioxidant capacity and reduce oxidative stress.

This study also evaluated the expression of key mediators of the UPR-PERK pathway (GRP78, PERK, eIF2α, ATF4, CHOP and NRF2). Endoplasmic reticulum (ER) stress is triggered by the accumulation of misfolded proteins, activating the UPR, in which GRP78 plays a central role as a molecular chaperone [32].

The HCD group showed increased expression of GRP78, PERK (*Eif2ak3*), eIF2α, ATF4, CHOP (*Ddit3*) and *Nfe2l2* compared to the SD group, indicating activation of ER stress and the UPR pathway. These findings are consistent with previous studies linking obesity and metabolic stress with ER dysfunction [33,34]. GRP78 plays a dual role, acting as a protective chaperone in early stages of ER stress but contributing to pathological processes when overexpressed chronically [35,36]. In the present study, the reduction in GRP78 expression in the HCD + EA group may reflect attenuation of ER stress rather than direct inhibition, consistent with the improved metabolic and oxidative profile.

PERK activation regulates protein synthesis and contributes to oxidative stress control. Increased PERK expression in the HCD group suggests impaired ER homeostasis, whereas its reduction in the HCD + EA group indicates partial recovery of ER function [37]. Similarly, the increased expression of eIF2α, ATF4, CHOP in the HCD group reflects activation of stress and apoptotic pathways, while their normalization in the HCD + EA group suggests attenuation of ER stress.

The PERK/eIF2α/ATF4 axis plays a key role in hepatic steatosis by converting endoplasmic reticulum stress into an adaptive response. Under lipid overload, PERK phosphorylates eIF2α at Ser51, enabling the selective translation of ATF4 and the regulation of autophagic genes such as LC3-II and ATG5, which are essential for lipophagy and hepatic lipid clearance [14]. The progression of hepatic steatosis is closely linked to a failure in the maintenance of the defense mechanism (adaptive UPR) [38].

This study reveals that ellagic acid (EA) acts as a therapeutic modulator, directly participating in ER signaling. Treatment with EA restores the phosphorylation status at Ser51, although a high-calorie diet induces chronic stress that depletes the eIF2α response. EA ensures the constant production of ATF4 and the maintenance of LC3-II-mediated lipophagy by preserving this phosphorylation state. EA prevents hepatocyte from transitioning from an adaptive signaling stage to a maladaptive phenotype, which is characterized by the activation of the CHOP-dependent terminal proapoptotic cascade.

In conclusion, the eIF2α molecular sensor converts ER stress into a lipophagy response through phosphorylation of the Ser51 residue, which selectively activates ATF4 and the LC3/ATG5 clearance genes [14]. MAFLD is characterized by a disruption of this phosphorylated state [38], which prevents the formation of autophagosomes from the ER [39]. According to our research, ellagic acid prevents the transition to hepatic apoptosis and restores eIF2α phosphorylation, which in turn restores LC3-II biogenesis for lipid clearance [40].

NRF2 plays a key role in antioxidant defense. The increased expression of *Nfe2l2* observed in the HCD group likely represents a compensatory adaptive response to elevated oxidative stress. However, despite this increase, the HCD group still exhibited elevated MDA levels and reduced antioxidant capacity, suggesting that NRF2 induction alone was insufficient to restore redox homeostasis. In contrast, the EA group showed lower *Nfe2l2* expression together with reduced lipid peroxidation and improved antioxidant enzyme activity, including SOD, CAT, and GPx. These findings suggest that the antioxidant effects of EA may reduce the cellular demand for sustained NRF2 induction by attenuating oxidative stress and PERK-associated ER stress signaling. Since NRF2 activity is predominantly regulated at the post-transcriptional level through KEAP1-mediated mechanisms, changes in mRNA expression do not necessarily reflect reduced antioxidant function.

Additionally, alternative pathways, such as SIRT1/FOXO signaling, may contribute to the antioxidant effects of ellagic acid [41]. The results suggest that ellagic acid modulates oxidative stress and ER stress pathways, contributing to improved metabolic and hepatic outcomes under hypercaloric diet conditions.

Overall, these findings indicate that the beneficial effects of ellagic acid are more evident under conditions of metabolic stress, supporting its potential role as a modulator of oxidative and ER stress rather than a general metabolic regulator.

## 4. Materials and Method

### 4.1. Materials

Ellagic acid (E2250-10G) purchased from Sigma Aldrich. The diets were prepared in the laboratory and the following products were purchased for their preparation: Cholesterol (from sheep wool, ≥92.5% (GC), powder # C8503), choline chloride (C7527-1K Sigma Aldrich, USA), DL-methionine (Sigma Aldrich C59518), lactic acid casein (NZMP, Fonterra, New Zealand), microcrystalline cellulose pH 101 (Lot no. CI509078), pregelatinized starch 515 (Ingredion, USA), sucrose (food grade), Vitamin Mix (AIN-93-VX), Mineral Mix (AIN-93-G), butter (Cremeria Americana, Mexico), extra virgin olive oil (Grupo Deoleo, Spain), powdered fructose (Quimabi, Mexico). For the acclimatization week, commercial feed Lab diet Rodent 5001 was used.

### 4.2. Diet Composition

The diets were formulated and prepared in the polymer laboratory of the Escuela Nacional de Ciencias Biológicas-IPN, Zacatenco. Table 3 shows the composition of each diet. The nutritional composition (Table 4) of the standard diet (SD) was 29.3% total protein, 57.1% carbohydrates, and 13.6% fat, corresponding to an energy value of 3.3 kcal/g, while the composition of the high-calorie diet (HCD) was 17% protein, 41.9% carbohydrates, and 41.2% lipids with a total energy value of 4.6 kcal/g.

### 4.3. Rat Model and Treatment

**Figure 7:**
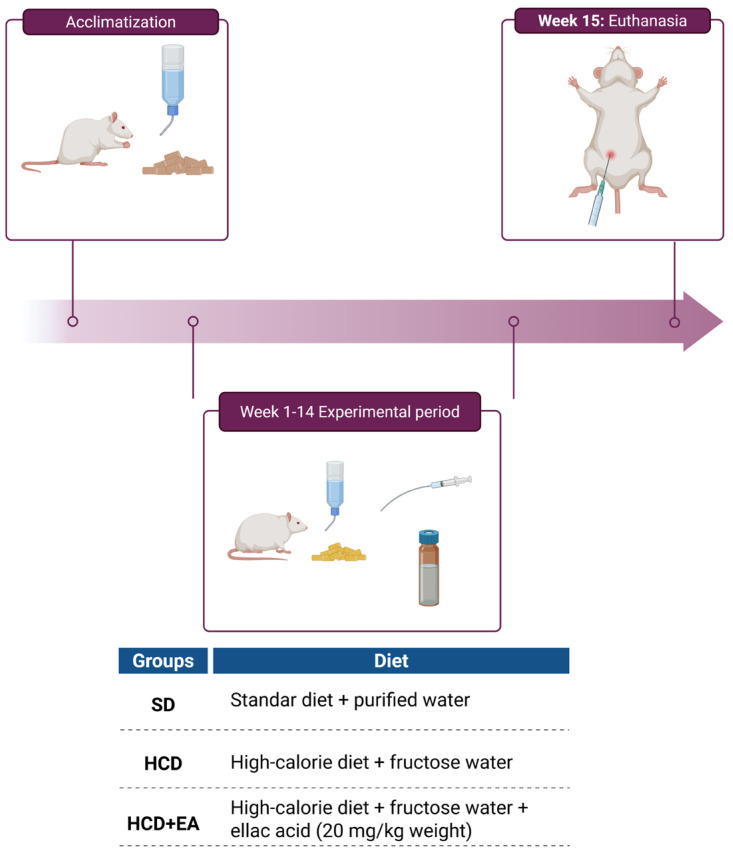
Timeline of the rat model trial. The animals spent a week acclimatizing at the facility, with food and water *ad libitum*. Subsequently, from week 2 to week 15, they were divided into three groups of eight rats each, assigned two different types of diet, and included ellagic acid treatment in one of the groups.

Male Wistar rats weighing 160 ± 20 g, purchased from Círculo ADN S.A. de C. V.- Mexico City, were housed in stainless steel cages under controlled conditions of temperature (22 ± 2 °C), humidity (40–60%), and 12 h light-dark cycles. The rats had a one-week adaptation period to the animal facility prior to the experiment with water and food *ad libitum*. After the first week, the animals were weighed and randomly divided into three groups (*n* = 8), according to the diet and treatment assigned: standard diet (SD) and water *ad libitum*; high-calorie diet (HCD) plus 10% fructose solution (100 g fructose/L water) and the third group were fed a high-calorie diet and fructose water, but with daily treatment with ellagic acid (20 mg/kg body weight) (HCD + EA) using Tween 80 (1%) as a vehicle for homogeneous dispersion of the compound; the dose which was used was chosen based on others reported in previous studies [42,43] and determined based on Table S1 [44,45,46,47,48,49,50,51,52] included in the Supplementary Materials. The selection of 20 mg/kg was based on the observation that beneficial effects on steatosis and oxidative stress have been consistently reported in the 10–90 mg/kg range. We chose an intermediate dose to maximize efficacy while minimizing potential toxicity and applied a single treatment regimen to maintain homogeneity and comparability with prior studies. Groups SD and HCD received water: Tween 80 solution instead of the treatment (Figure 7). This experimental design is consistent with previous reports demonstrating that high-fat and high-calorie diets reproduce the endoplasmic reticulum stress and UPR activation observed in human MAFLD/MASH, thus representing a translationally relevant model. In addition to body weight and serum lipids, histopathological analysis of liver tissue was included to confirm the onset of MAFLD in the present study. The experimental protocol was conducted in accordance with the Code of Ethics for Animal Studies of the Escuela Nacional de Ciencias Biológicas (ENCB-IPN, Mexico City, Mexico) ENCB/CICUAL/013/2025 AUT–B–B05219;–052 (submitted on 8 April 2025) and the Guide for the Care and Use of Laboratory Animals (NOM–062–ZOO–1999).

### 4.4. Determination of Weight and Food and Water Consumption

Changes in the animals’ body weight were monitored every third day during the fifteen weeks of experimentation. Food and water intake were measured daily.

Food consumption was measured by weighing 60 g of food daily and then weighing the leftover food the following morning to quantify daily intake. The food was then replaced with 60 g so that the animals always had plenty of food available. In the case of water, 100 mL of water was added to the drinking troughs, and the following day, the remaining water was measured to quantify the rats’ consumption, and 100 mL of water was added again.

### 4.5. Lipid and Biochemical Profile

At the end of the experimental period (week 14), the animals were fasted for 12 h, after which blood glucose levels were measured in blood extracted from the tail vein using a glucometer (ABBOT, USA). The rats were then anesthetized with pentobarbital (120 mg/kg body weight).

Serum was extracted from each rat, and total HDL cholesterol, LDL, and VLDL, as well as triglycerides, insulin, glycosylated hemoglobin, and levels of aspartate aminotransferase, alanine aminotransferase, were measured using automated photometry/low-pressure liquid chromatography with Autokem II, Controlab equipment.

### 4.6. Processing and Evaluation of Liver Histological Sections

Biological liver samples obtained from experimental animals were fixed in formalin buffer solution and embedded in paraffin. Histological sections 3-μm thick were prepared and stained with hematoxylin and eosin. A morphological evaluation was performed on a Zeiss Primostar 3 microscope, Germany.

Histological parameters and phenotypic quantification included hepatocyte swelling (0 = none, 1 = few swollen cells, 2 = many cells/prominent swelling), macrovesicular and microvesicular fatty change (1 minimal, 2 mild, 3 moderate, 4 marked, 5 severe; D-diffuse, Z-zonal: C-centrilobular, P-periportal, M-mezogonial, and affected hepatocytes: 0 = <5%, 1 = 5–33%, 2 = 33–66%, 3 = >66%), lobular inflammation (F-focal/M-multifocal, D-diffuse, C-centrilobular, M-mediolobular, P-periportal, PB-peribiliary, N-neutrophilic, L-lymphocytic, H-histiocytic, foci per optical field x200: 1 = <2, 2 = 2, 3 = >4).

### 4.7. RNA Extraction and Polymerase Chain Reaction

The extraction of RNA started from 200 mg of liver tissue and was carried out using the TRIzoltm Reagent (Invitrogen, USA; 15596026) with MagNA Lysier Green Beads (Roche, SA; 03358941001). The tubes were placed in a MagNA Lyser Instrument to obtain the lysate, and then the RNA was purified following the instructions in the TRIzol(tm) Reagent User Guide provided by the manufacturer. To quantify the RNA concentration (OD-260) and purity (OD-260/OD-280), 1 µL of RNA was deposited on the NanoDrop 2000 spectrophotometer (Thermo Scientific, USA; ND2000).

RNA integrity was determined in 1% agarose (BioRad 1613102, Hercules, CA, USA) gel electrophoresis was performed using a BioRad horizontal electrophoresis system (BioRad 1704467, Hercules, CA, USA). The reaction was carried out with M-MLV Reverse Transcriptase (Promega, USA; M1701) to proceed with cDNA synthesis. Finally, the cDNA was used for qPCR analysis using the Rotor-Gene Q QIAGEN with miRCURY LNA SYBR Green PCR Kit (QIAGEN, Germany; 339347) with 0.5 µM concentrations of oligonucleotides corresponding to the mRNAs of the UPR-PERK pathway genes Grp78, Eif2a, Eif2ak3, Nfe2l2, Ddit3, and Atf4; and β-actin as the reference gene. All the oligos were designed with the oligo 3.0 program (Table 5). The PCR conditions were as follows: 95 °C for 10 min, 40 cycles at 95 °C for 10 s and 60 °C for 60 s. Relative gene expression was calculated by the ΔΔCt method using expression data of the β-actin gene as the normalizer.

### 4.8. Quantification of UPR-Perk Pathway Proteins

To quantify proteins in the UPR-PERK pathway, the following ELISA kits were used: GRP78 (Cat. No. MBS1600255) MyBiosourse, USA; PERK (Cat. No. LS-F55870) LSBio, USA; eIF2α (Cat. No. MBS021800); ATF4 (Cat. No. ABIN6953421) Antibodies Online, USA; CHOP (Cat. No. MBS3808179) MyBiosourse; NRF2 (Cat. No. MBS012148) MyBiosourse, and the manufacturer’s instructions were followed.

### 4.9. Antioxidant Capacity by the ORAC Method in the Liver

Antioxidant capacity was determined using the method described by Quek et al. 2021 [53]. The tissue was homogenized in phosphate buffer at pH 7.4 to process the samples in a 1:10 ratio. The homogenates were then centrifuged for 30 min at 15,000× *g* at 4 °C, and the supernatant was collected.

To measure the samples, 20 µL of the supernatant was added to a dark 96-well plate, and 20 µL of 6-hydroxy-2,5,7,8-tetramethylchromane-2-carboxylic acid (trolox) (1000 µM) instead of the sample or 20 µL of phosphate buffer for the blank. Subsequently, 120 µL of fluorescein (96 nM) was added, followed by 60 µL of 2,2′-azobis (2-amidino-propane) dichlorohydrate (AAPH) (12 mM).

The loss of fluorescence was read every 2 min for 2 h using a microplate reader (SYNERGY H1, Bio Tek, Winooski, VT, USA), with an excitation wavelength of 485 nm and an emission wavelength of 515 nm.

The ORAC value was calculated using the following equation and the results were expressed as TEAC values (µM Trolox/g dry weight sample).

ORAC value = [(AUC sample − AUC control)/(AUC Trolox − AUC control)]·FD

where

AUC sample = area under the sample curve.

AUC control = area under the control curve.

AUC Trolox = area under the curve using Trolox as a standard sample.

FD = Sample dilution factor.

### 4.10. Superoxide Dismutase Enzyme Activity

This assay was performed using the technique described by Valderrama Díaz 2014 [54], with some modifications. The tissues were homogenized in a cold buffer solution (100 mM Tris-HCl, 0.1 mM EDTA, and 0.1% Triton X-100 (*v*/*v*), pH 7.8, in a 1:4 (*w*/*v*) ratio. The homogenate was centrifuged at 15,000× *g* for 30 min at 4 °C. The supernatants were then collected, and a 50 µL aliquot was taken. To bring the samples to 25% ethanol, 12.5 µL of ethanol was added. The aliquots were centrifuged at 12,000× *g* for 20 min at 4 °C, and the supernatant was stored for future use.

Two mixtures were prepared: MTT 1.2 mM and EDTA 0.05 mM; hydroxylamine 67 mM and calcium carbonate 182 mM.

Twenty µL of the samples were added to each well of the microplate and 20 µL of 25% ethanol was added for the blank. Subsequently, 70 µL of the MTT + EDTA mixture and 110 µL of the hydroxylamine + calcium carbonate mixture were added. Finally, the plate was read at 560 nm in a microplate reader (SYNERGY H1, BioTek, Winooski, VT, USA). Readings were taken every 15 min for 3 h.

The calculations to determine SOD activity were obtained using the following formula.

SOD = (∆ white/∆ABS sample/min)/[ ] Protein (mg/mL)

### 4.11. Catalase Enzymatic Activity

The catalase assay was performed following the technique of Valderrama Díaz, 2014 [54].

The tissue was homogenized in phosphate buffer at pH 7.4 to process the samples in a 1:10 ratio. The homogenates were then centrifuged for 30 min at 15,000× *g* at 4 °C, and the supernatant was collected.

A reaction medium containing 60 mM H_2_O_2_, 1 M Tris HCl, and 5 mM EDTA at pH 8 was prepared.

In quartz cells, 10 µL of the sample and 990 µL of the reaction medium were placed. For the blank, 10 µL of the buffer used to homogenize the samples was taken. Absorbance readings were immediately taken at 240 nm at 10 s intervals for 1 min at 25 °C.

Catalase activity was expressed in U/mg of protein, i.e., one unit of activity was defined as the amount of enzyme required to transform one µmol of substrate per minute. The following formula was used for the calculation:

CAT = ((∆ABS/10s) × Total volume × dilution)) × 60 s/(Protein mg/mL × extinction coefficient)

To calculate catalase’s activity, the molar extinction coefficient of H_2_O_2_ was used: 39.58 M^−1^ × cm^−1^. The terms were expressed in mg of protein.

### 4.12. Aldehyde Dehydrogenase Activity

The assay was performed using the Cayman Chemical (USA) aldehyde dehydrogenase activity assay kit (700800) according to the manufacturer’s specifications.

### 4.13. Lipid Peroxidation Assay

The lipid peroxidation assay, which measures MDA, is commonly performed using the thiobarbituric acid reactive substances assay, which forms a pink MDA-TBA complex. In this study, liver tissue samples were mixed with water (0.5 g of tissue and 1 mL of water), homogenized, and then 2 mL of Tris-HCl (150 mM)-TCA (15%)-TBA (0.375%) solution was added. The samples were incubated at 37 °C for 30 min, and the samples were centrifuged at 3000 rpm for 10 min. Then, 1 mL of the supernatant was taken and added to a quartz cell to measure in a Thermo Scientific Genesys 10S UV-Vis spectrophotometer, USA at 532 nm. A 1:10 dilution of the homogenate of each sample was made to determine proteins using Bradford. Activity was expressed in nmoles of MDA/mg of protein.

### 4.14. Determination of Glutathione Peroxidase (GPx) Activity

Glutathione peroxidase (GPx) activity was determined using an assay based on the oxidation of reduced glutathione (GSH) and its subsequent quantification with Ellman’s reagent. The technique was performed following the methodology of Sattar et al., 2024 [55].

A phosphate buffer pH 7.0 was prepared by combining 100 mM solutions of anhydrous dibasic sodium phosphate (Na_2_HPO_4_) and 100 mM monobasic potassium phosphate (KH_2_PO_4_). Initially, 50 mL of 100 mM Na_2_HPO_4_ was taken and the pH was adjusted to 7.0 by gradually adding 100 mM KH_2_PO_4_. Subsequently, 50 mL of the adjusted buffer was taken and EDTA (0.372 mM) and sodium azide (0.6501 mM) were added. The solution was made up to 100 mL with the same pH 7.0 buffer and stored at 4 °C until use.

Tissue samples were homogenized at a ratio of 100 mg of tissue per mL of PBS. The homogenate was centrifuged at 15,000× *g* for 30 min, and the supernatant obtained was used for analysis.

The Ellman’s reagent was prepared by dissolving 0.01 g of tribasic sodium citrate and 0.002 g of 5,5′-dithiobis-(2-nitrobenzoic acid) (DTNB) in distilled water and making up to 10 mL. The solution was protected from light until use. A 1.5 mM hydrogen peroxide (H_2_O_2_) solution was also prepared in distilled water and measured to 10 mL.

For the standard curve, a 500 µM GSH stock solution was used, from which dilutions of 400, 300, 250, 200, 150, 100, 75, and 50 µM were prepared. In a microplate, 100 µL of each standard was placed in duplicate and 10 µL of distilled water was added. The plates were incubated for 10 min at 37 °C and the absorbance at 405 nm was recorded every minute for 10 min. Subsequently, 65 µL of 100 mM phosphate buffer and 35 µL of Ellman’s reagent were added, incubated for 3 min, and the final reading was taken at 405 nm.

For the determination in samples, 10 µL of the supernatant and 100 µL of 500 µM GSH were placed in a microplate. A control without GSH addition was prepared for each sample. After incubation for 10 min at 37 °C, 40 µL of 1.5 mM H_2_O_2_ was added and incubated again for 10 min at 37 °C, recording the absorbance at 405 nm every minute for 10 min. The reaction was stopped by adding 65 µL of 100 mM phosphate buffer and 35 µL of Ellman’s reagent. After 3 min of incubation, the final reading was taken at 405 nm.

A blank assay was included, which contained no sample or GSH; instead, 110 µL of distilled water was added. The blank was subjected to the same experimental procedure described for the samples.

### 4.15. Determination of GSH and GSSG

For sample preparation, 300 mg of tissue was weighed and 1 mL of 0.143 M sodium phosphate buffer supplemented with 0.005 M EDTA, adjusted to pH 8.0, was added. The tissue was homogenized and then centrifuged at 15,000× *g* for 30 min. The supernatant obtained was collected and kept cold until processing.

The following solutions were prepared: metaphosphoric acid (MPA) by dissolving 5 g in 50 mL of distilled water; 4 M triethanolamine (TEAM), which was used within 4 h of preparation (stable at 25 °C); and a 10% o-phthaldialdehyde (OPA) solution in absolute methanol, kept at 4 °C and protected from light until use.

The samples were deproteinized, 1 mL of the supernatant obtained previously was mixed with 1 mL of freshly prepared MPA, vortexed, and allowed to stand for 5 min in the dark at room temperature. Subsequently, it was centrifuged at 15,000× *g* for 30 min and the supernatant was recovered. To 1 mL of this supernatant, 50 µL of 4 M TEAM was added, vortexed, and kept cold. The neutralized samples were divided into two aliquots of 500 µL each for the determination of reduced glutathione (GSH) and oxidized glutathione (GSSG).

For GSH quantification, 100 µL of deproteinized samples were placed in a microplate and 40 µL of 10% OPA was added. The mixture was incubated for 15 min in the dark at room temperature. Fluorescence was measured using λexcitation = 350 nm and λemission = 420 nm.

To determine oxidized glutathione (GSSG), a solution of 0.004 M N-ethylmaleimide (NEM) in phosphate buffer pH 8.0 was prepared. A solution of 0.1 N NaOH (pH 12) in 100 mL of distilled water was also prepared. Subsequently, 100 µL of deproteinized sample was mixed with 100 µL of 0.004 M NEM and incubated for 1 h in the dark at room temperature. After this time, the mixture was diluted with 800 µL of 0.1 N NaOH to obtain a final volume of 1 mL. Finally, 100 µL of this dilution was taken and 40 µL of 10% OPA was added. The mixture was incubated for 15 min in the dark at room temperature and the fluorescence was measured using λexcitation = 350 nm and λemission = 420 nm.

### 4.16. Statistical Analysis

Data are expressed as mean ± SEM. Comparisons among experimental groups (SD, HCD and HCD + EA) for variables measured at a single time point were performed using one-way analysis of variance (ANOVA) followed by Tukey’s post hoc test for multiple comparisons. Variables measured over time, including body weight, food intake and water intake, were analyzed using two-way ANOVA with repeated measures (group × time), followed by Tukey’s post hoc test. Differences were considered statistically significant at *p* ≤ 0.05. All analyses were performed using GraphPad Prism software (version 8.0.2).

## 5. Conclusions

The hypercaloric diet activated the UPR pathway in response to endoplasmic reticulum stress. Reticulum stress caused an increase in the expression of Grp78, Eif2ak3 (PERK), Eif2α, Ddit3 (CHOP), Atf4, and Nfe2l2 (Nrf2) genes in both liver and epididymal adipose tissue. The administration of ellagic acid generated a protective effect by modulating the expression of UPR PERK pathway genes, so it could be considered a potential candidate as a hepatoprotective agent.

The HCD + EA group ameliorated endoplasmic reticulum stress, and the biochemical profile compared to the HCD group. In addition to regulating endoplasmic reticulum stress, the HCD + EA group also showed regulation in lipid and biochemical profile levels compared to the HCD group. Their glucose levels, total cholesterol, HDL cholesterol, VLDL cholesterol, triglycerides, insulin, and glycated hemoglobin showed a decrease.

Ellagic acid helped preserve liver tissue structure, reduce cellular vacuolization, and limit the progression of structural changes associated with diet-induced damage, indicating a protective response at both the biochemical and morphological levels.

The ellagic acid treatment helped preserve liver tissue structure, reduce cellular vacuolization, and limit the progression of structural changes associated with diet-induced damage, indicating a protective response at both the biochemical and morphological levels.

The high-fat diet induced a state of oxidative stress characterized by decreased antioxidant capacity, alterations in antioxidant enzymes, and increased lipid peroxidation, evidenced primarily by decreased ORAC, increased MDA, and the redox index. Although some endogenous antioxidant systems, such as glutathione, showed minimal changes, the set of biomarkers indicates an alteration in the oxidative state associated with diet-induced metabolic damage. Treatment with ellagic acid demonstrated a significant hepatoprotective effect by partially restoring antioxidant capacity, increasing the activity of the antioxidant enzymes SOD and CAT, promoting the detoxification of aldehydes through increased ALDH activity, and reducing lipid peroxidation to levels close to those of the SD group. Likewise, the normalization of the redox index and the preservation of the glutathione system suggest better maintenance of intracellular oxidative balance.

This mechanistic proposal suggest that a high-calorie diet induces a systemic metabolic disturbance characterized by adipose tissue expansion, increased oxidative stress, and activation of ER stress, contributing to the development of hepatic abnormalities consistent with the early stages of metabolic dysfunction-associated steatotic liver disease. It has been reported that the excess of free fatty acids resulting from obesity and visceral adiposity promotes mitochondrial dysfunction and overproduction of ROS, favoring lipotoxicity, inflammation and activation of the UPR [56].

Within the UPR, the PERK-mediated pathway plays a central role in cellular adaptation to oxidative stress. PERK activation induces the phosphorylation of eIF2α, decreasing overall protein synthesis and promoting the selective translation of ATF4. Although this mechanism is initially cytoprotective, prolonged activation promotes the expression of DDIT3, leading to inflammation, apoptosis, and the progression of liver damage [57,58]. Consistent with this, the HCD group showed increased gene expression of *Grp78*, *Eif2ak3*, *eIF2α*, *Atf4*, Ddit3 and NF-κB, accompanied by increased MDA and decreased ORAC and antioxidant enzymes, suggesting sustained activation of cellular stress mechanisms.

Treatment with ellagic acid demonstrated a significant antioxidant effect, as evidenced by a reduction in lipid peroxidation and a partial recovery of SOD, CAT, and GSH levels. Recent studies have confirmed that ellagic acid exerts hepatoprotective effects by regulating oxidative stress, inflammation, and lipid metabolism. A recent review reported that ellagic acid reduces liver damage and metabolic dysfunction by modulating antioxidant, inflammatory, and mitochondrial pathways related to NAFLD/NASLD [59]. Furthermore, a clinical trial published in 2025 showed that ellagic acid supplementation in patients with NAFLD significantly reduced MDA, triglycerides, LDL, and liver enzymes, supporting its therapeutic potential as a modulator of oxidative stress [52].

Several studies have suggested that ellagic acid exerts its antioxidant effects by activating NRF2 and dissociating the KEAP1/CUL3 complex, thereby promoting the expression of antioxidant genes [60,61]. However, in the present study, a decrease in NF-κB was observed in the treated group compared to the HCD group. This finding could be explained by a reduced need for compensatory antioxidant activation secondary to the partial restoration of redox balance. It is known that NRF2 is predominantly regulated at the post-translational level, and its activity depends more on stabilization and nuclear translocation than on sustained increases in gene expression. Therefore, the observed reduction in Nfe2l2 could reflect a decrease in oxidative stress rather than a loss of antioxidant protection.

Additionally, the model proposed in the graphical abstract suggests a possible interaction between ellagic acid and metabolic regulators such as SIRT1 and FOXO1/FOXO3. Recent studies have shown that polyphenolic compounds can activate the AMPK/SIRT1/FOXO axis, promoting the expression of antioxidant enzymes and improving mitochondrial function and hepatic β-oxidation [56]. Although these pathways were not directly evaluated in the present study, they could help explain the partial recovery of the antioxidant system observed following treatment with ellagic acid.

Taken together, the proposed mechanism suggests that ellagic acid may exert a hepatoprotective effect primarily by restoring redox homeostasis and attenuating endoplasmic reticulum stress, thereby reducing the sustained activation of the UPR-PERK/eIF2α/ATF4/CHOP pathway. These findings suggest that the beneficial effects of ellagic acid are more closely related to the modulation of oxidative stress and hepatic cytoprotection than to a direct reduction in body weight.

## Figures and Tables

**Table 1:** Effect of a high-calorie diet and ellagic acid on the lipid and biochemical profile.

Parameters	SD	HCD	HCD + EA
Initial body weight (g)	212.2 ± 9.1 ^a^	217.3 ± 9.7 ^a^	232.8 ± 8.1 ^a^
Final body weight (g)	561.7 ± 11.4 ^a^	631 ± 8 ^b^	605.7 ± 11.6 ^ab^
Body weight gain (g)	349.4 ± 5.06 ^a^	413.7 ± 16.4 ^b^	372.8 ± 12.7 ^ab^
Energy intake (kcal/day)	99.6 ± 1 ^a^	142 ± 1 ^b^	131.5 ± 2 ^c^
Glucose (mg/dL)	107.2 ± 1.2 ^a^	130 ± 4 ^b^	116.4 ± 4.9 ^ab^
Total cholesterol mg/dL	74.2 ± 11 ^a^	104.6 ± 7.4 ^a^	92.4 ± 5.9 ^a^
HDL cholesterol (mg/dL)	24.8 ± 3.9 ^a^	21.8 ± 1.6 ^a^	22.8 ± 2.5 ^a^
LDL cholesterol (mg/dL)	29.8 ± 8 ^a^	36.2 ± 6.3 ^a^	42 ± 8.6 ^a^
VLDL cholesterol (mg/dL)	19 ± 4.6 ^a^	37.2 ± 3.4 ^b^	17.6 ± 4.6 ^a^
Triglycerides (mg/dL)	102 ± 25.1 ^a^	194.6 ± 15.6 ^b^	93.4 ± 27.9 ^a^
Insulin (ng/mL)	6.8 ± 0.6 ^a^	11.8 ± 1 ^b^	8.1 ± 0.3 ^ab^
Glycosylated hemoglobin (%)	6.3 ± 0.3 ^a^	9.4 ± 1 ^b^	7.6 ± 0.4 ^ab^

Data are presented as means ± SEM (*n* = 8). SD: standard diet; HCD: high-calorie diet; HCD + EA: high-calorie diet with ellagic acid treatment. Values with different letters indicate statistically significant differences between groups (*p* ≤ 0.05; one-way ANOVA followed by Tukey’s post hoc test).

**Table 2:** Changes in endogens biomarkers of oxidative stress in liver tissue due to ellagic acid treatment.

Group	ORAC (µmol TE/g)	SOD µmol/min/mg Protein	CAT µmol/min/mg Protein	GPx UAE: µM GSSG/min/Protein mg	GSH µg/Tissue mg	GSSG µg/Tissue mg	Redox Index µg/Tissue mg	MDA (nmol MDA/mg Protein)	ALDH (ALDH Activity Units
SD	301.4 ± 18.45 ^a^	0.132 ± 0.07 ^a^	0.012 ± 0.01 ^a^	5.17 ± 0.4 ^a^	17.49 ± 1.03 ^a^	4.93 ± 0.18 ^a^	0.22 ± 0.006 ^a^	0.067 ± 0.006 ^a^	0.44 ± 0.20 ^a^
HCD	241.9 ± 9.39 ^b^	0.117 ± 0.07 ^a^	0.008 ± 0.002 ^a^	4.48 ± 0.4 ^a^	16.44 ± 0.44 ^a^	5.61 ± 0.16 ^a^	0.25 ± 0.007 ^a^	0.177 ± 0.040 ^b^	0.75 ± 0.34 ^a^
HCD + EA	295.7 ± 12.84 ^a^	0.220 ± 0.02 ^b^	0.025 ± 0.01 ^a^	7.19 ± 0.65 ^b^	18.70 ± 1.04 ^a^	4.97 ± 0.27 ^a^	0.218 ± 0.02 ^a^	0.075 ± 0.018 ^a^	2.617 ± 0.58 ^b^

Data are presented as mean ± SEM (*n* = 8). SD: standard diet; HCD: high-calorie diet; HCD + EA: high-calorie diet with ellagic acid. Different letters indicate statistically significant differences between groups (*p* ≤ 0.05; one-way ANOVA followed by Tukey’s post hoc test). ORAC, oxygen radical absorbance capacity; SOD, superoxide dismutase; CAT, catalase; GPx, glutathione peroxidase. ORAC assesses antioxidant capacity by measuring the inhibition of peroxyl radical-induced oxidation; SOD catalyzes the conversion of the superoxide anion into molecular oxygen and hydrogen peroxide; CAT breaks down hydrogen peroxide into water and oxygen; GPx catalyzes the oxidation of reduced glutathione to oxidized glutathione using peroxides as a substrate. GSH: the primary intracellular antioxidant in its reduced form, involved in neutralizing reactive oxygen species. GSSG: the oxidized form of glutathione produced following the oxidation of two GSH molecules. Redox index: the ratio of reduced to oxidized glutathione (GSSG/GSH + GSSG), used as an indicator of cellular redox status. MDA: produced as a result of the reaction of free radicals with lipids, which can serve as a marker of oxidative stress. ALDH: catalyzes the oxidation of aldehydes to carboxylic acids, contributing to cellular detoxification.

**Table 3:** Diet composition.

Ingredients	SD Concentration (g/kg)	HCD Concentration (g/kg)
Sucrose	60	340
Oil/Butter	51	210
Casein	246	195
Starch	420	140.5
Cellulose	60	50
Mineral Mix ^1^	43	43
Vitamin Mix ^2^	15	15
DL-Methionine	3	3
Choline	2	2
Cholesterol	0.196	1.5
kcal/g	3.3	4.6

Note: ^1^ Mineral mix (AIN-93-G-MX); ^2^ Vitamin mix (AIN-93-VX). SD: standard diet; HCD: high-calorie diet.

**Table 4:** Macronutrients in diets.

	SD (%)	HCD (%)
Protein	29.3	17
Carbohydrates	57.1	41.9
Lipids	13.6	41.2

**Table 5:** Sequence of primers used for RT-qPCR.

Gene	Forward Primer Sequence	Reverse Primer Sequence	NCBI Reference Sequence
*Gpr78*	GCAGTTGCTCACGTGTCTTG	CTGACCCAGCTTTTCCCCAA	NM_013083.2
*Eif2a*	AAAACCTGGCTGTAGTGGCA	TCAGAGATCCACCTGCCTCT	NM_001399818.1
*Eif2ak3*	GTGACTGCAATGGACCAGGA	CTTGTCCCGTGTGTGTAGCA	NM_031599.2
*Nfe2l2*	CCCCTGGAAGTGTCAAACAGAA	CGACAGAGGCTGTACTGTATCC	NM_001399173.1
*Ddit3*	TCGCCTTTGAGACAGTGTCC	AGGACCTCCTGCAGATCCTC	NM_001109986.1
*Atf4*	CCAATTGGCCATCTCCCAGA	CAGGGAAGAGGCTGCAAGAA	NM_024403.2
*β-actin*	CTAAGGCCAACCGTGAAAAG	TACATGGCTGGGGTGTTGA	NM_031144.3

## Data Availability

Will be provided upon request to the authors.

## Supplementary Materials

The following supporting information can be downloaded at: https://www.mdpi.com/article/10.3390/ijms27104491/s1.

## Abbreviations

The following abbreviations are used in this manuscript:

MAFLD	Metabolic dysfunction-Associated Fatty Liver Disease
NAFLD	Non-alcoholic fatty liver disease
MAFL	Metabolic dysfunction-Associated Fatty Liver
MASH	Metabolic dysfunction-Associated Steatohepatitis
UPR	Unfolded protein response
NAFL	Hepatic steatosis
NASH	Steatohepatitis
FFA	Free fatty acids
IHTG	Intrahepatic triglycerides
ROS	Reactive oxygen species
IRE1	Inositol-requiring enzyme 1
ATF6	Activator transcription factor 6
GRP78	Glucose Regulated Protein 78
PERK	Protein Kinase R-like Endoplasmic Reticulum Kinase
eIF2α	Phosphorylates eukaryotic initiation factor 2α
ATF4	Activating Transcription Factor 4
CHOP	DDIT3 transcription factor
NRF2	Nuclear factor erythroid 2-related factor 2
MAPK	Mitogen-activated protein kinases
SREBP-1	Sterol regulatory element binding protein-1
HCD	High-calorie diet
EA	Ellagic acid
LDL	Low-density lipoprotein
VLDL	Very low-density lipoprotein
HDL	High-density lipoprotein
ALDH	Aldehyde dehydrogenase
TG	Triglycerides
ORAC	Oxygen radical absorbance capacity
SOD	Superoxide dismutase
CAT	Catalase
GPX	Glutathione peroxidase
GSH	Reduced glutathione
GSSG	Oxidized glutathione
MDA	Malondialdehyde
Cul3	Cullin-3
PPAR-α	Peroxisome proliferator-activated receptor alpha
