# Supplementary material for: Modulation of mRNA Expression of Biomarkers in the UPR-PERK Pathway by Ellagic Acid in Metabolic Dysfunction-Associated Fatty Liver Disease
Source: Int J Mol Sci. 2026 May 17;27(10):4491. doi: 10.3390/ijms27104491 (PMC13206915; doi:10.3390/ijms27104491)
Supplement: Supplementary file 1 [file ijms-27-04491-s001.zip › ijms-4260733-supplementary.pdf]

## Supplementary material

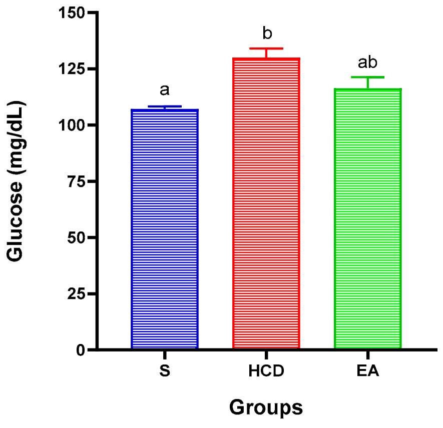


a)

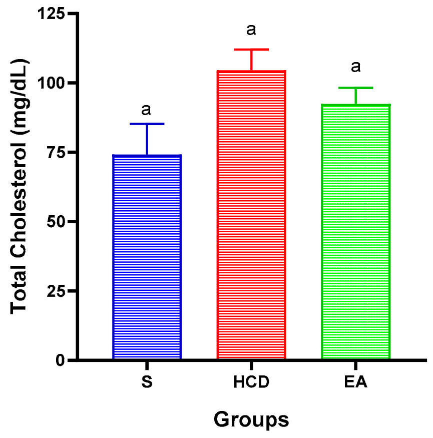


b)

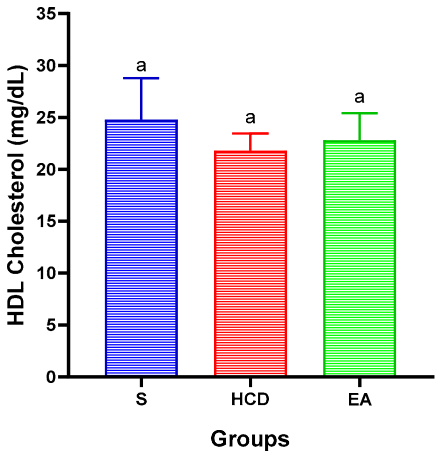


c)

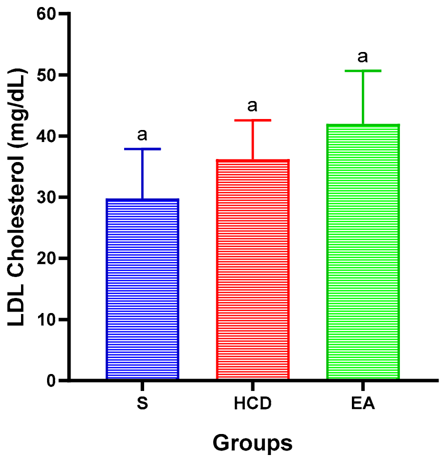


d)

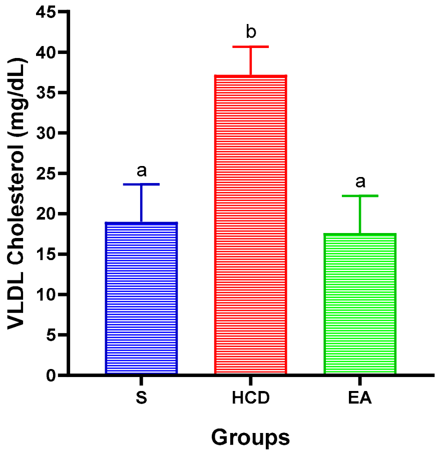


e)

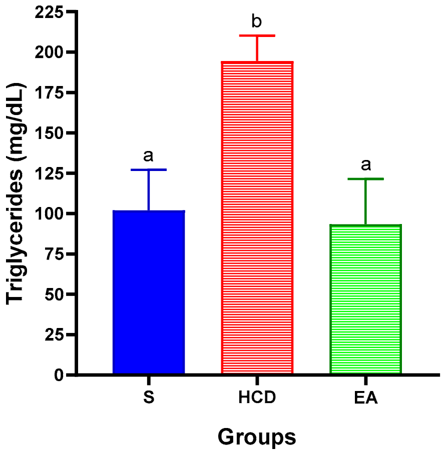


f)

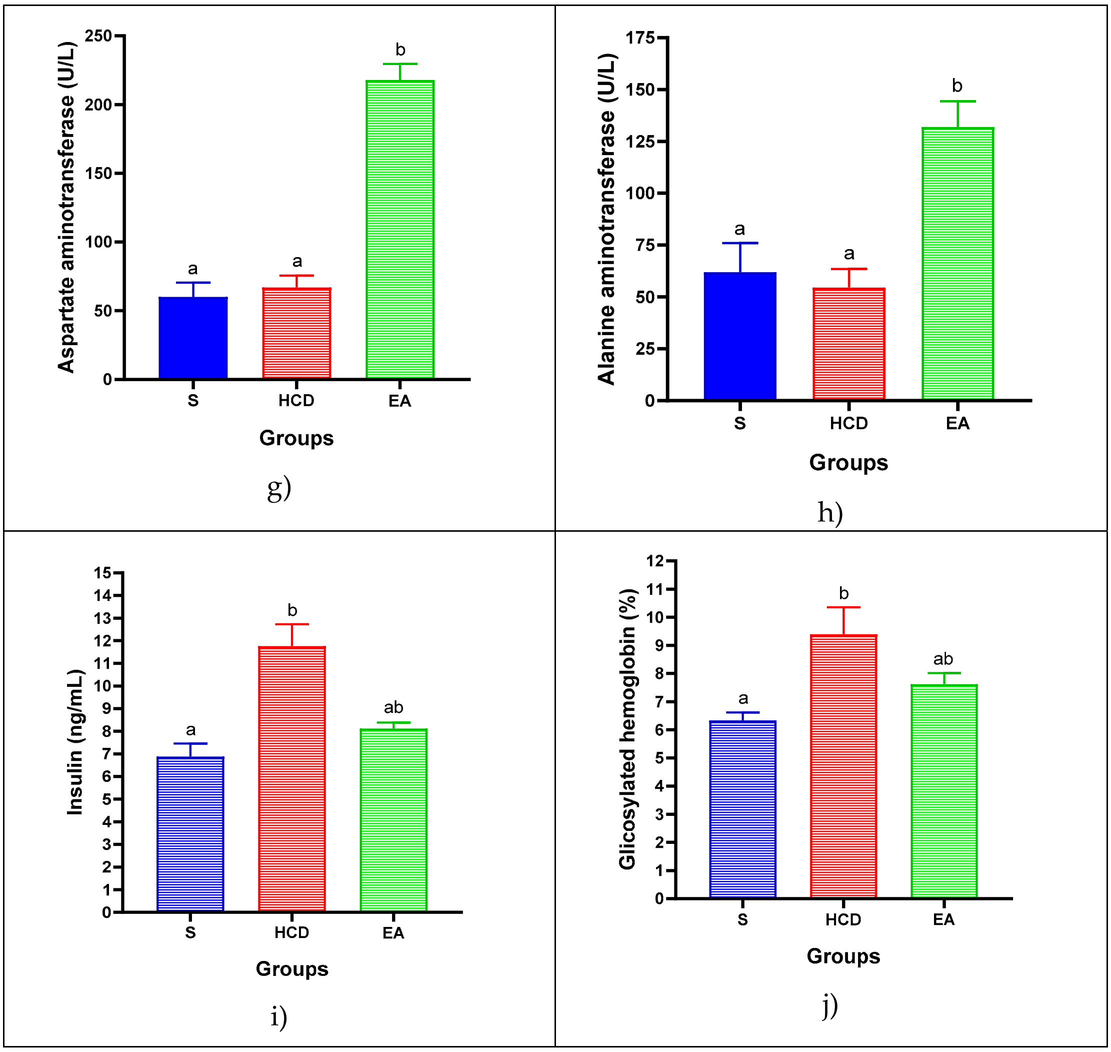


Figure S1. Effect of high-calorie diet and ellagic acid on lipid and biochemical profile. a) Glucose level results. The HCD group shows a tendency to increase compared to the S group. Meanwhile, the EA treatment shows a tendency to decrease compared to the HCD group, suggesting that treatment with ellagic acid could help reduce the increase in glucose caused by the high-calorie diet. b) Total cholesterol levels did not show significant differences between the groups ($p > 0.05$). However, a trend toward higher levels was observed in the HCD group compared to the S group ($p=0.07$). c) HDL cholesterol levels did not show significant differences between the experimental groups ($p>0.05$). d) LDL cholesterol levels remained unchanged in all experimental groups, with no significant differences detected ($p>0.05$). e) The HCD group shows a tendency to increase VLDL cholesterol levels compared to the S group, and the EA group shows a significant reduction compared to the HCD group. f) The HCD group tends to increase triglyceride levels compared to the S group, while the EA group decreases them to levels very similar to those of the S group. g) The HCD group shows a tendency to decrease aspartate aminotransferase levels compared to the S group, and the EA group shows a significant increase compared to the HCD group. h) The HCD group also shows a tendency to decrease alanine aminotransferase levels compared to the S group, while the EA group shows a

tendency to increase. i) The HCD group shows a significant increase in insulin levels compared to the S group, while the EA group shows a decrease in levels compared to the HCD group, even to levels similar to the S group. j) The HCD group shows a tendency to increase glycosylated hemoglobin levels compared to the S group, and the EA group shows a tendency to decrease compared to the HCD group.

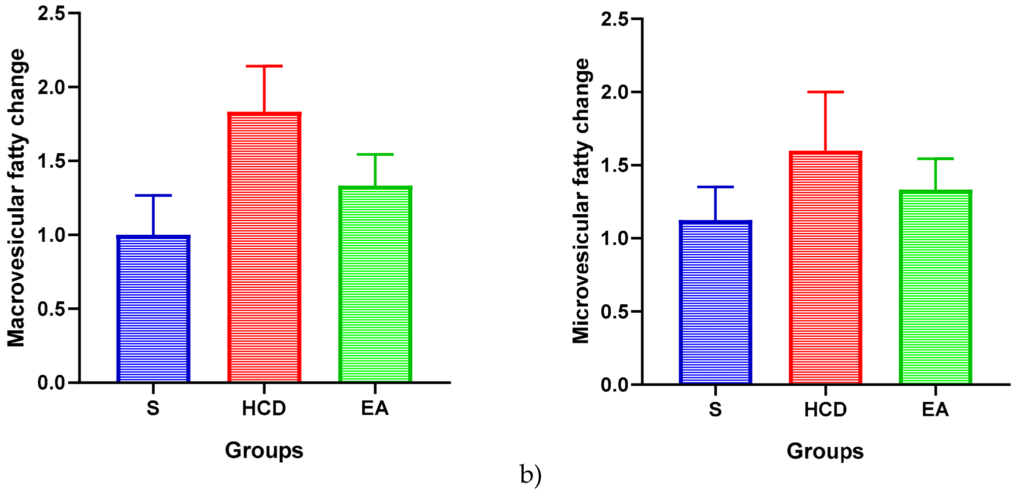


a) b)

**Figure S2** a) shows the level of macrovesicular damage to liver tissue. The HCD group showed a level of damage classified as mild to moderate, while the EA group showed minimal damage. Graph b) shows the level of microvesicular damage, classifying the HCD group as mild to moderate and the EA group as minimal.

Table S1 summarizes the available studies on the use of ellagic acid in models of liver damage. None of the included studies have directly evaluated markers in the PERK pathway of UPR (PERK, eIF2$\alpha$, ATF4, CHOP), leaving room for further research on the effect of ellagic acid on this pathway. As can be seen from the data presented in this table, there is a reduction in hepatic steatosis from doses of 10 mg/kg body weight to 90 mg/kg body weight, so a dose of 20 mg/kg body weight was selected.

**Table S1.** Studies on the effect of ellagic acid on liver inflammation and steatosis.

| Dosage | Model | Results | References |
|---|---|---|---|
| 30, 60 y 90 mg/kg | Female Wistar rats. | It significantly reduced elevated AST and ALT levels.<br>It restored cholesterol, free fatty acid, triglyceride, and phospholipid levels.<br>Histopathological evaluation showed a reduction in cellular degeneration and inflammatory infiltration in the liver. | (Devipriya et al., 2008) [44] |
| 0,1% | KK-Uny mice on a high-fat diet as a model for obese type 2 diabetes. | It improved the lipid profile and hepatic steatosis; decreased serum resistin; and up-regulated the mRNA expression of the apoa1, ldlr, cpt1a, and ppar$\alpha$ genes in the liver. | (Yoshimura et al., 2013) [45] |

| | | | |
|---|---|---|---|
| 10 y **30** mg/kg | Rats | It attenuated weight gain, glucose intolerance, and white adipocyte hypertrophy induced by a high-fat diet.<br>An improvement in the serum lipid profile and a significant reduction in hepatic steatosis were observed. | (Wang et al., 2019) [46] |
| 25 μM | 3T3-L1 preadipocyte cells | It decreased lipid accumulation (lipogenesis) through gene regulation of glucose transporter type (GLUT) 4 and adiponectin.<br>It inhibits TNF-$\alpha$, nitric oxide synthase (iNOS), interleukin 6 (IL-6), and monocyte chemotactic protein-1. | (Cisneros-Zevallos et al., 2020) [47] |
| 50 mg/kg | Wistar rats with streptozotocin-induced type 1 diabetes, which generates a model of non-alcoholic hepatic steatosis. | Reduced in liver: ROS, MDA, TNF-$\alpha$, IL-6, NF-κB p65 nuclear activity. Increased Nrf-2 nuclear activity, GSH, SOD. Modulation of gene expression related to lipid synthesis and degradation through AMPK signaling activation. | (ALTamimi et al., 2021) [48] |
| 15 mg/kg | Male Wistar rats, induced with a diet high in fructose at 20% in water (8 weeks) | It reduces levels of uric acid, glucose, insulin, serum lipids; lipogenic enzymes (ACL, FAS, SREBP-1c); MDA and XO; and increases GSH.<br>Histology revealed reduced steatosis and inflammation in the liver. | (Elseweidy et al., 2022) [49] |
| 50 mg/kg | C57BL/6 mice on a high-fat diet (HFD) | Reduces TG, TC, LDL-C; increases HDL-C; improves glucose tolerance; reduces hepatic steatosis; increases PPAR$\gamma$ and lipid genes; reduces MDA; increases SOD, CAT, GSH-Px | (Li et al., 2024) [50] |
| 10, **25**, 50 mg/kg | Rats | Reduces necroptosis, increases autophagy, improves lipid profile. Reduces levels of proinflammatory cytokines (such as TNF-$\alpha$ and IL-6) and increases anti-inflammatory cytokines (IL-4, IL-10). Increases the activity of antioxidant enzymes (SOD, CAT, GR, GPx) and reduces MDA in the liver. It had a significant effect at 25 mg/kg. | (Li et al., 2025) [51] |
| 180 mg/day | Randomized, double-blind, placebo-controlled clinical trial. | Significant decrease in ALT, AST, ALP, and GGT liver enzyme levels; decrease in TG and LDL-c; increase in antioxidant capacity; decrease in malondialdehyde (MDA), decrease in levels of C-reactive protein (CRP), IL-6, and TNF-$\alpha$, as well as improvement in blood glucose, insulin, insulin resistance (IR), and hemoglobin A1c (HbA1c) levels. | (Mighani et al., 2025) [52] |
